# Supplementary material for: A self-attention model for inferring cooperativity between regulatory features
Source: Nucleic Acids Res. 2021 May 5;49(13):e77. doi: 10.1093/nar/gkab349 (PMC8287919; doi:10.1093/nar/gkab349)
Supplement: gkab349_Supplemental_File [file gkab349_supplemental_file.pdf]

Supplementary Methods

Data collection and processing

A. Experiment 1: Simulated dataset

In this experiment, we simulated DNA sequences using random sampling from a distribution of [0.27, 0.23, 0.23, 0.27] for A, C, G, and T respectively as used for a similar dataset generated by Greenside et al. [1]. We generated 120,000 sequences each with a length of 200 bp. Similar to Greenside et al. [1], we randomly embedded instances of the motifs of both ELF1 and SIX5 transcription factors in 40,000 of the total sequences. This was our positive set of examples where we essentially simulated interactions between the aforementioned motifs. In the negative set (80,000 sequences), we embedded instances of either ELF1 or SIX5 in a sequence (but not both). Moreover, we embedded instances of the AP1 and TAL1 motifs across all examples. The motifs for the four transcription factors were obtained from Kheradpour et al. [2].

TF Database information

To map CNN filters to motifs of known TFs, we used TomTom with a custom TF database (MEME format) containing PWMs of the four transcription factors: SIX5, ELF1, AP1, and TAL1.

B. Experiment 2: TAL-GATA ChIP-peaks

Here we followed the same strategy described in DFIM [1]: ChIP-Seq peaks were downloaded for the three TFs TAL1, GATA1, and GATA2 from the ENCODE [4] database in the K562 cell line (hg19 genome assembly and annotations). For the chromatin accessibility data, we downloaded processed DNase I Hypersensitive Sites (DHSs) from the ENCODE database for the corresponding cell line. The raw data (bed files) is available at DFIM github repository. Next, every ChIP-Seq peak for the three transcription factors was searched for an overlap with DHSs in the K562 cell line. If an overlap was found, the sequence of the ChIP-Seq peak was extended 500 bp upstream and downstream from its center. This served as a positive set in our binary classification problem. For the negative set, we randomly sampled 80,000 examples from all K562 DHSs that didn't overlap a ChIP-Seq peak for any of the three transcription factors.

TF Database information

In this experiment, we used two TF databases: the first one was a custom motif file with PWMs of TAL1, GATA1, and GATA2 transcription factors. This was because we wanted to directly compare our model to DFIM [1] where Greenside et al. measured interactions between the aforementioned transcription factors. The second reference was the entire CISBP TF database [3] that we used in order to infer other TF interactions within the ChIP-Seq peaks.

C. Experiment 3: Human promoter DHSs

In this experiment, we used DHSs overlapping gene promoter regions across the entire human genome. We used the pipeline described by Kelley et al. in Basset [5]: DHSs were downloaded for 164 human immortalized cell lines from the ENCODE [4] and ROADMAP [6] consortia

(more information on the samples can be found in their paper and accompanied supplement). These regions of open chromatin were merged if they overlapped more than 200 bp. Finally, every DHS was extended to a length of 600 bp around its center. Kelley et al. [5] used DHSs across the entire genome however, we selected only those which overlapped the human promoter regions. To do that, we defined promoter as a region of 1000 bp upstream of the transcription start site (TSS) of a gene—Ensemble based hg19/GrCh37 reference and annotations were used. The final dataset had 20,613 genomic sequences of the corresponding DHSs (that overlapped the human promoters). The targets in this case were either a single label or multiple labels, corresponding to the164 cell lines in which the DHSs were observed.

TF Database information

In motif analysis (and later in the TF interactions), we used the human CISBP transcription factor database [3].

D. Experiment 4: Genome-wide arabidopsis regions of open chromatin

Here we designed a similar experiment as described in the previous section. The dataset was constructed using the same procedure described above as used by Kelley et al. [5]. We used regions of open chromatin: DHSs and ATAC-Seq based Transposase Hypersensitive Sites (THSs), across the entire arabidopsis genome using TAIR10 annotations. We used the following publicly available datasets (GEO accession numbers provided):

1. **For DHSs:** GSE53322, GSE53324, GSE53323, GSE46987, GSE34318
2. **For THSs:** GSE89346, GSE85203, GSE101940, GSE116287, GSE101482

We ended up with 88,245 examples in our final dataset across 36 different samples. Note that peaks occurring in multiple biological samples were merged.

TF Database information

Here we used the DAP-Seq based arabidopsis transcription factors database [7].

Limitations of the TomTom motif comparison tool

We used the TomTom tool from the MEME suite [8] to map CNN filters to motifs of known transcription factors. In some cases, we observed the match to be dubious despite the tool assigning it a significant p-value. This is shown in Supplementary Figure F4 for two of our CNN filters matching the known TF motifs of HOXA2 and ZNF263 in the human CISBP database [3].
By default, TomTom uses *Pearson correlation* for comparing motifs. However, we obtained better results using the *Euclidean* distance.

Availability of TomTom results

For the four experiments, the TomTom motif matching results are available at our GitHub repository: https://github.com/fahadahaf/satori/tree/master/data/TomTom_files

Details on the multi-head self-attention layer

The input to the multi-head self-attention (MHA) layer is a $d \times R$ matrix where d is the length of the input sequence after the max-pooling operation—which is the last step in the convolutional layer—and R is the dimensionality of the input which equals:

1. Number of CNN filters when the recurrent layer is absent (see hyperparameter *CNN_filters* in Table S1).
2. Size of the RNN hidden dimension when the recurrent layer is present (see hyperparameter *RNN_hiddensize* in Table S1).

For an individual head, the attention output (see *equation 8* in the main text) is a $d \times h$ matrix where h is the size of the individual attention head, defined by the hyperparameter *singlehead_size* in Table S1. For k individual attention heads (for the value of k, see hyperparameter *num_multiheads* in Table S1), we concatenate the outputs and end up with a $d \times kh$ matrix. Next, the concatenated matrix (Z_m) is linearly transformed using the following:

$$M = Z_m^\top W_m,$$

where W_m is the associated weight matrix of dimensions $kh \times p$, and p is the size of the multi-head linear transformation, defined by the hyperparameter *multihead_size* in Table S1.

Next, the matrix M is summed across the columns and standardized, leading to a p-dimensional vector, which is passed to the fully connected layer. As mentioned in the main text, we find that this step is not only computationally efficient but also leads to better model accuracy in comparison to flattening the attention layer output. Note that the hyperparamter *readout_strategy* in Table S1 can be used to determine whether to do the aforementioned step (parameter value = "normalize") or flatten (parameter value = "flatten") the matrix M.

Supplementary Figures

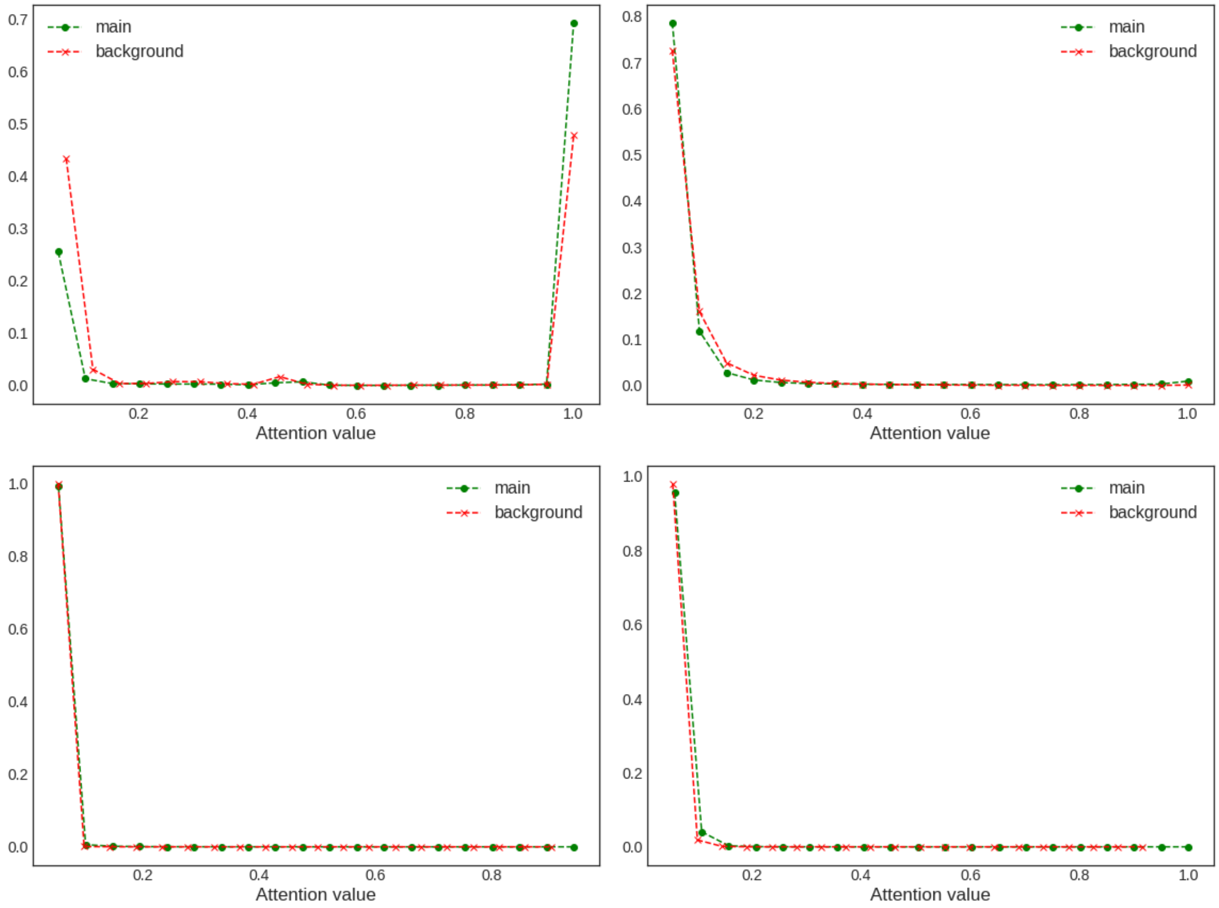

Figure F1: Distribution of the attention values for the test and the background sets are shown for the four experiments: simulated dataset (top-left), TAL-GATA ChIP peaks (top-right), human promoter DHSs (bottom-left) and arabidopsis accessible chromatin (bottom-right). The actual frequencies (y-axis) are normalized by total sizes of the test and background sets. This figure helps in selecting the appropriate attention cutoff, one of the parameters of SATORI. We use a default value of 0.10, although the model produces results at different thresholds.

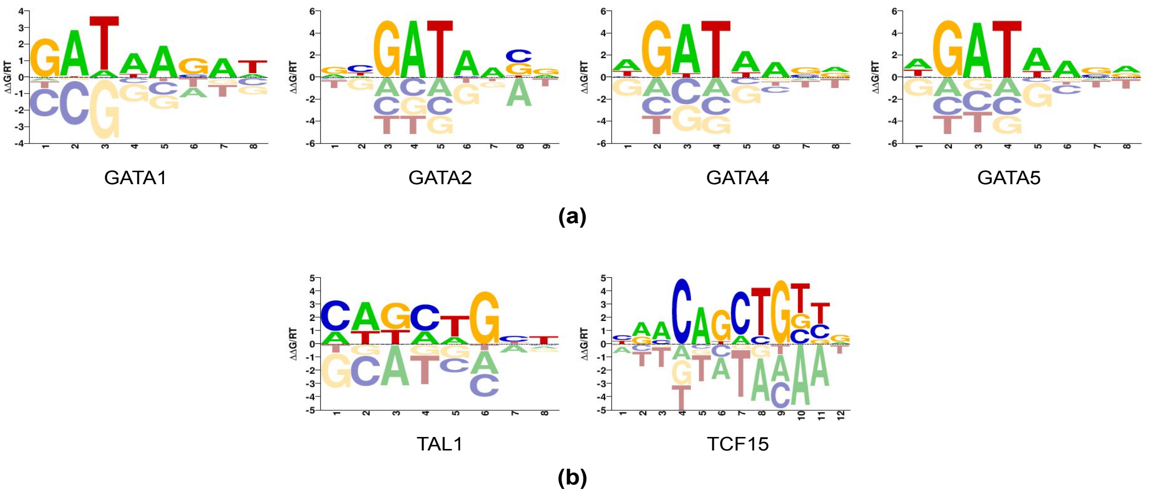

Figure F2: Similarities between motifs of GATA variants (a). Similarly, TAL1 and TCF15, both belonging to the bHLH family, have very similar motifs (CAGCTG consensus) (b).

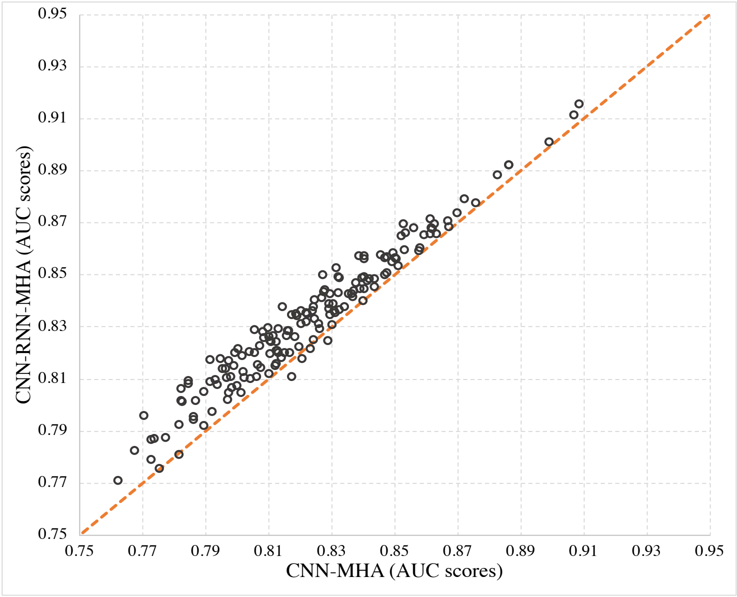

Figure F3: AUC scores for DHSs in human promoters across 164 cell types, achieved by the two model variants. Each circle represents performance on detecting DHSs in that cell line.

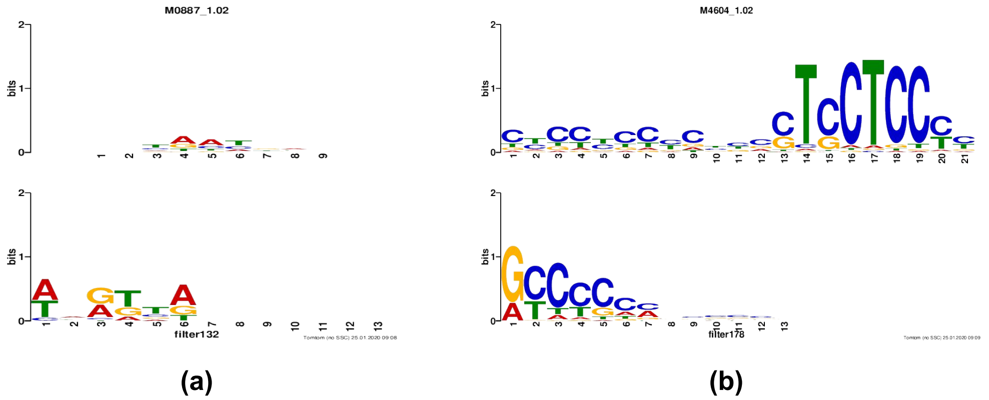

Figure F4: Limitations of the TomTom motif comparison tool. Matches shown here are detected as statistically significant (q-value < 0.01) for both (a) HOXA2 and (b) ZNF263. The top row depicts the gold standard motif in the CISBP database, and the bottom row shows the CNN filter/motif.

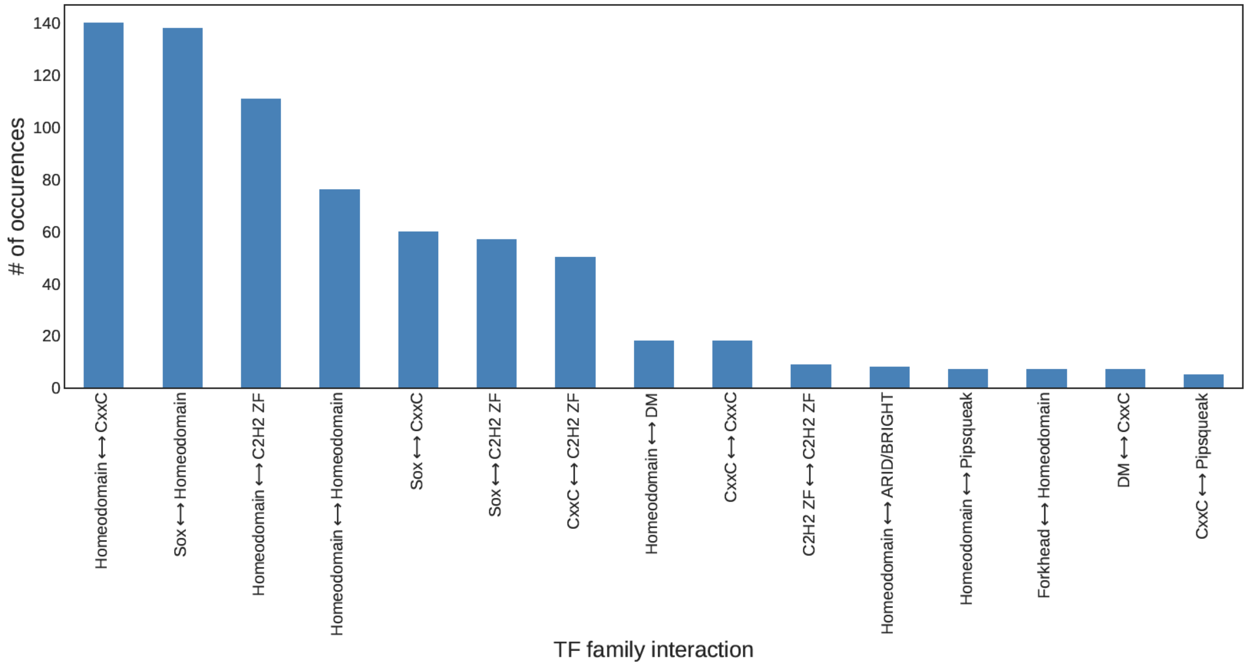

Figure F5: The most frequent interacting transcription factor families in human promoter regions.

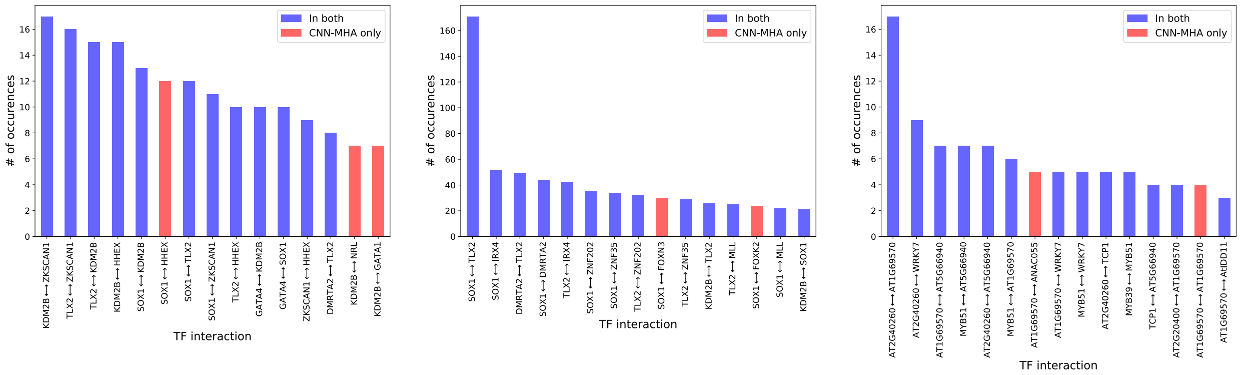

(a) (b) (c)

Figure F6: Common interactions in the top predictions of the two architectures: CNN-MHA and CNN-RNN-MHA. Interactions predicted using the CNN-MHA model are sorted by frequency. Those predicted by both methods are shown in blue, and ones predicted only by CNN-MHA are shown in red. Top predictions are shown for the TAL-GATA dataset (a) the human promoter dataset (b), and the genome-wide arabidopsis dataset (c).

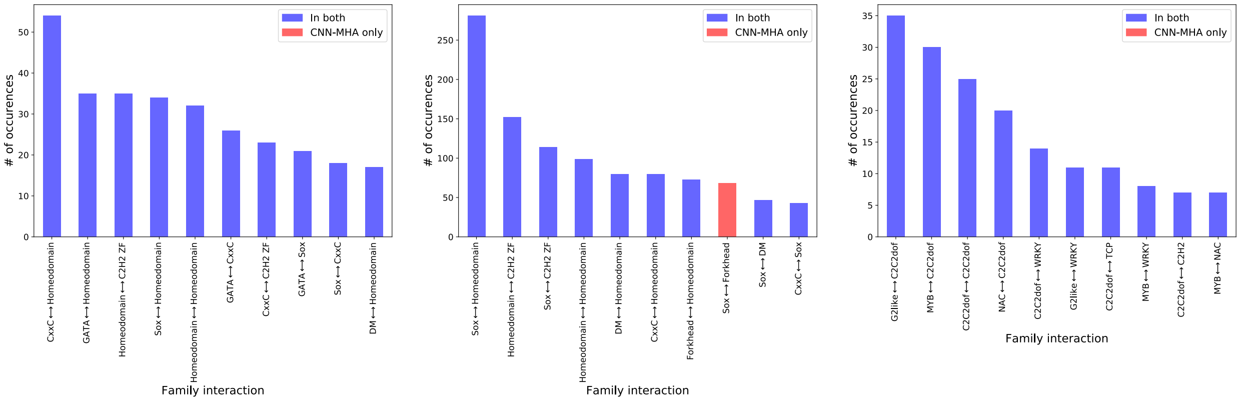

(a) (b) (c)

Figure F7: Common TF family interactions in the top predictions of the two architectures: CNN-MHA and CNN-RNN-MHA. Top predictions are shown for the TAL-GATA dataset (a) the human promoter dataset (b), and the genome-wide arabidopsis dataset (c).

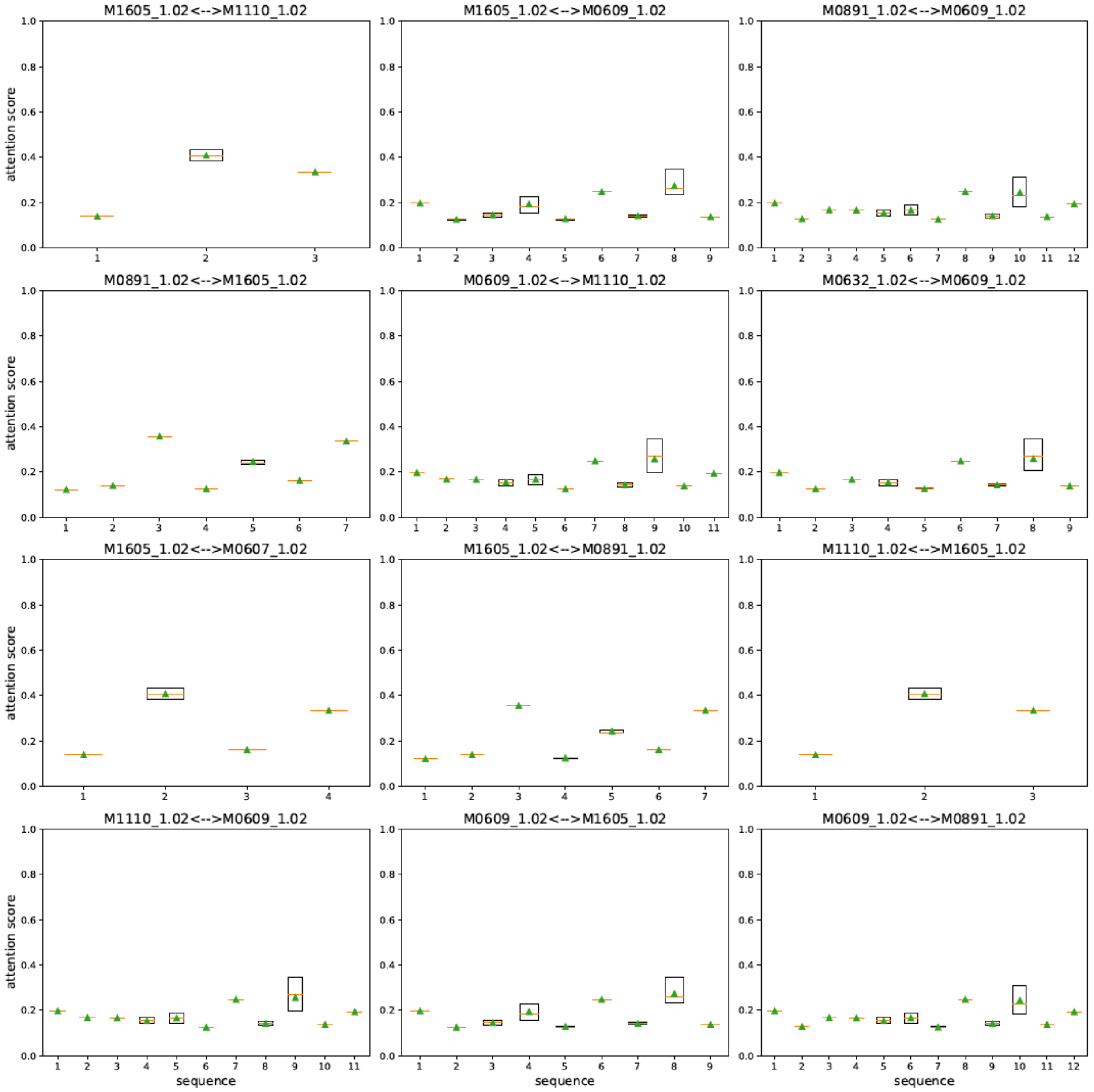

Figure F8: Distribution of attention scores (box plots) for twelve TF-TF interactions, each with multiple matching filters across all human promoter test sequences in which they appear.

Supplementary Tables

Table S1: List of neural network hyperparameters common across the four experiments.

Hyperparameter	type	Description
input_channels	int	Number of original input channels [Default: 4, for DNA]
num_multiheads	int	Number of heads in the multi-head attention [Default: 8]
singlehead_size	int	Size of the single attention head [Default: 32]
multihead_size	int	Size of the final attention after concatenation [Default: 100]
use_pooling	bool	Whether to use max pooling in the attention layer [Default: False]
pooling_val	int	Size of the max pooling window in the attention layer [Default: 6]
readout_strategy	str	Read-out strategy of the attention layer [Default: "normalize"]
use_RNN	bool	Whether to use the RNN layer [Default: True]
CNN1_useexponential	bool	Whether to use exponential activation in the convolution [Default: False]
CNN_filters	int	Number of filters in the convolutional layer [Default: 200]
CNN_filtersize	int	Filter size in the convolution [Default: 13]
use_CNNpool	bool	Whether to use max pooling in the convolutional layer [Default: True]
CNN_poolsize	int	Size of the max pooling window in the convolutional layer [Default: 6]
RNN_hiddensize	int	Size of the RNN/LSTM unit [Default: 100]
batch_size	int	Batch size in training and testing [Default: 256]
num_epochs	int	Number of training epochs [Default: 50]

Table S2: Summary of all significant interactions in the simulated/toy dataset. Our model is able to recover multiple interactions involving SIX5 and ELF1 TF motifs. We also provide the actual CNN filter interactions in the first column, named based on the total number of filters in the convolutional layer.

Filter interaction	TF motif interaction	Adjusted p-value
filter033↔filter170	SIX5↔ELF1	1.47E-42
filter170↔filter181	ELF1↔SIX5	1.50E-39
filter033↔filter111	SIX5↔ELF1	1.12E-20
filter111↔filter181	ELF1↔SIX5	3.17E-22
filter033↔filter091	SIX5↔ELF1	2.44E-18
filter091↔filter181	ELF1↔SIX5	2.14E-12
filter033↔filter055	SIX5↔ELF1	2.64E-08
filter055↔filter181	ELF1↔SIX5	3.84E-04
filter019↔filter033	ELF1↔SIX5	1.35E-07
filter019↔filter181	ELF1↔SIX5	2.48E-12
filter091↔filter199	ELF1↔SIX5	2.82E-03

Table S3: All significant interactions between TAL1 and GATA transcription factors. Note that in this case, a custom TF database was used containing motifs for TAL1, GATA1, and GATA2. In case of TAL1, other TFs (LYL1, NHLH2, and TAL2) also shared the same binding site motif and hence are mentioned here.

TF A	TF B	Adjusted p-value
GATA2	LYL1, **TAL1**, NHLH2, TAL2	4.37E-24
GATA2	LYL1, **TAL1**, NHLH2, TAL2	8.18E-21
LYL1, **TAL1**, NHLH2, TAL2	GATA2	5.71E-19
LYL1, **TAL1**, NHLH2, TAL2	GATA2	1.54E-16
LYL1, **TAL1**, NHLH2, TAL2	GATA2	8.68E-11
GATA2	LYL1, **TAL1**, NHLH2, TAL2	3.92E-10
LYL1, **TAL1**, NHLH2, TAL2	GATA2	3.27E-08
LYL1, **TAL1**, NHLH2, TAL2	GATA2	3.28E-08
GATA2	LYL1, **TAL1**, NHLH2, TAL2	5.66E-05
LYL1, **TAL1**, NHLH2, TAL2	GATA2	6.76E-04
LYL1, **TAL1**, NHLH2, TAL2	GATA2	4.51E-03
LYL1, **TAL1**, NHLH2, TAL2	GATA2	4.66E-03
LYL1, **TAL1**, NHLH2, TAL2	GATA2	4.72E-03
LYL1, **TAL1**, NHLH2, TAL2	GATA2	5.41E-03

Table S6: A list of known TF interactions identified by our model in the human promoter dataset. TRRUSTv2 [9] and HIPPIE v2.0 [10] were used as references for known interactions. The level of significance (adjusted p-value) assigned by our model to each interaction is provided in the last column.

Motif interaction	TF1 family	TF2 family	adjusted p-value	Database
DNMT1↔LCOR	CxxC	Pipsqueak	3.38E-29	TRRUSTv2
DNMT1↔EGR1	C2H2 ZF	CxxC	1.30E-22	
EGR1↔LCOR	C2H2 ZF	Pipsqueak	3.58E-08	
FOXK2↔EGR1	C2H2 ZF	Forkhead	1.09E-04	
DNMT1↔KDM2B	CxxC	CxxC	1.61E-33	HIPPIE v2.0
DNMT1↔LCOR	CxxC	Pipsqueak	3.38E-29	
HHEX↔TLX2	Homeodomain	Homeodomain	5.88E-23	
KDM2B↔LCOR	CxxC	Pipsqueak	2.36E-18	
KDM2B↔HHEX	Homeodomain	CxxC	5.24E-18	
FOXK2↔DNMT1	Forkhead	CxxC	3.63E-12	
HHEX↔ZNF32	Homeodomain	C2H2 ZF	1.24E-07	
SOX3↔EGR1	Sox	C2H2 ZF	3.06E-07	
FOXK2↔KDM2B	Forkhead	CxxC	2.07E-05	

Table S7: Summary of the number of unique statistically significant TF interactions reported by SATORI and FIS for the three real-world datasets.

Experiment	SATORI	FIS
TAL-GATA ChIP-Seq	172	284
Human promoters	123	168
Arabidopsis genome-wide	687	511

Table S4: A list of all unique TF interactions in the TAL-GATA ChIP-Seq peaks dataset. (appended to this supplement.)

Table S5: A list of all unique significant interactions in the human promoters. (appended to this supplement.)

References

1. Greenside, P., Shimko, T., Fordyce, P., & Kundaje, A. (2018). Discovering epistatic feature interactions from neural network models of regulatory DNA sequences. *Bioinformatics*, *34*(17), i629-i637.
2. Kheradpour, P., & Kellis, M. (2014). Systematic discovery and characterization of regulatory motifs in ENCODE TF binding experiments. *Nucleic acids research*, *42*(5), 2976-2987.
3. Lambert, S. A., Jolma, A., Campitelli, L. F., Das, P. K., Yin, Y., Albu, M., ... & Weirauch, M. T. (2018). The human transcription factors. *Cell*, *172*(4), 650-665.
4. Gerstein, M. B., Kundaje, A., Hariharan, M., Landt, S. G., Yan, K. K., Cheng, C., ... & Min, R. (2012). Architecture of the human regulatory network derived from ENCODE data. *Nature*, *489*(7414), 91-100.
5. Kelley, D. R., Snoek, J., & Rinn, J. L. (2016). Basset: learning the regulatory code of the accessible genome with deep convolutional neural networks. *Genome research*, *26*(7), 990-999.
6. Kundaje, A., Meuleman, W., Ernst, J., Bilenky, M., Yen, A., Heravi-Moussavi, A., ... & Amin, V. (2015). Integrative analysis of 111 reference human epigenomes. *Nature*, *518*(7539), 317-330.
7. Bartlett, A., O'Malley, R. C., Huang, S. S. C., Galli, M., Nery, J. R., Gallavotti, A., & Ecker, J. R. (2017). Mapping genome-wide transcription-factor binding sites using DAP-seq. *Nature protocols*, *12*(8), 1659.
8. Bailey, T. L., Johnson, J., Grant, C. E., & Noble, W. S. (2015). The MEME suite. *Nucleic acids research*, *43*(W1), W39-W49.
9. Han, H., Cho, J. W., Lee, S., Yun, A., Kim, H., Bae, D., ... & Lee, S. (2018). TRRUST v2: an expanded reference database of human and mouse transcriptional regulatory interactions. *Nucleic acids research*, *46*(D1), D380-D386.
10. Alanis-Lobato, G., Andrade-Navarro, M. A., & Schaefer, M. H. (2016). HIPPIE v2. 0: enhancing meaningfulness and reliability of protein–protein interaction networks. *Nucleic acids research*, gkw985.

Table S4: TAL-GATA ChIP unique TF interactions			
**filter_interaction**	**TF_Interaction**	**Family_Interaction**	**adjusted_pval**
filter96⟷filter104	DMRTA2⟷FOXN3	DM⟷Forkhead	0.016273919
filter104⟷filter129	DMRTA2⟷GATA5	DM⟷GATA	0.014186112
filter30⟷filter133	DMRTA2⟷GSX1	Homeodomain⟷DM	0.03069758
filter62⟷filter104	DMRTA2⟷HES2	DM⟷bHLH	0.002780668
filter30⟷filter160	DMRTA2⟷HHEX	Homeodomain⟷DM	0.027453357
filter104⟷filter153	DMRTA2⟷HOXA11	Homeodomain⟷DM	0.004447103
filter104⟷filter143	DMRTA2⟷KDM2B	DM⟷CxxC	5.79E-05
filter104⟷filter199	DMRTA2⟷PRKRIR	DM⟷THAP finger	0.015684653
filter30⟷filter57	DMRTA2⟷RFX7	DM⟷RFX	0.038614307
filter30⟷filter108	DMRTA2⟷RXRB	DM⟷Nuclear receptor	0.001270302
filter30⟷filter159	DMRTA2⟷SOX1	DM⟷Sox	0.032421057
filter61⟷filter104	DMRTA2⟷TCF15	DM⟷bHLH	0.005685621
filter30⟷filter196	DMRTA2⟷TLX2	Homeodomain⟷DM	0.026231892
filter78⟷filter104	DMRTA2⟷ZKSCAN1	C2H2 ZF⟷DM	0.04570174
filter17⟷filter30	DUXA⟷DMRTA2	Homeodomain⟷DM	0.002179452
filter17⟷filter96	DUXA⟷FOXN3	Homeodomain⟷Forkhead	7.49E-06
filter17⟷filter129	DUXA⟷GATA5	Homeodomain⟷GATA	0.008553442
filter17⟷filter133	DUXA⟷GSX1	Homeodomain⟷Homeodomain	0.001298725
filter17⟷filter62	DUXA⟷HES2	Homeodomain⟷bHLH	1.66E-05
filter17⟷filter160	DUXA⟷HHEX	Homeodomain⟷Homeodomain	0.0025193
filter17⟷filter153	DUXA⟷HOXA11	Homeodomain⟷Homeodomain	0.008288421
filter17⟷filter27	DUXA⟷IRX4	Homeodomain⟷Homeodomain	2.63E-05
filter17⟷filter143	DUXA⟷KDM2B	Homeodomain⟷CxxC	2.65E-10
filter17⟷filter199	DUXA⟷PRKRIR	Homeodomain⟷THAP finger	0.002570267
filter17⟷filter108	DUXA⟷RXRB	Homeodomain⟷Nuclear receptor	0.030577167
filter17⟷filter171	DUXA⟷SOX1	Homeodomain⟷Sox	0.012312924
filter17⟷filter61	DUXA⟷TCF15	Homeodomain⟷bHLH	1.93E-05
filter17⟷filter152	DUXA⟷TLX2	Homeodomain⟷Homeodomain	0.024489316
filter17⟷filter78	DUXA⟷ZKSCAN1	Homeodomain⟷C2H2 ZF	0.001731158
filter17⟷filter86	DUXA⟷ZNF202	Homeodomain⟷C2H2 ZF	0.014991929
filter96⟷filter133	FOXN3⟷GSX1	Homeodomain⟷Forkhead	0.004969243
filter96⟷filter153	FOXN3⟷HOXA11	Homeodomain⟷Forkhead	1.32E-05
filter96⟷filter143	FOXN3⟷KDM2B	Forkhead⟷CxxC	0.000622207
filter96⟷filter199	FOXN3⟷PRKRIR	Forkhead⟷THAP finger	0.001806896
filter96⟷filter108	FOXN3⟷RXRB	Forkhead⟷Nuclear receptor	0.019092169
filter96⟷filter159	FOXN3⟷SOX1	Forkhead⟷Sox	0.00264225
filter96⟷filter129	GATA5⟷FOXN3	GATA⟷Forkhead	3.41E-07
filter129⟷filter133	GATA5⟷GSX1	Homeodomain⟷GATA	0.004665757
filter129⟷filter160	GATA5⟷HHEX	Homeodomain⟷GATA	0.011465718
filter129⟷filter153	GATA5⟷HOXA11	Homeodomain⟷GATA	3.06E-05
filter129⟷filter198	GATA5⟷PAX2	GATA⟷Homeodomain,Paired box	0.010233609
filter89⟷filter129	GATA5⟷RARB	GATA⟷Nuclear receptor	0.037681423
filter84⟷filter139	GATA5⟷RARG	GATA⟷Nuclear receptor	1.03E-12

filter108⟷filter129	GATA5⟷RXRB	GATA⟷Nuclear receptor	0.007053379
filter84⟷filter154	GATA5⟷UNKNOWN	GATA⟷UNKNOWN	0.01304683
filter133⟷filter153	GSX1⟷HOXA11	Homeodomain⟷Homeodomain	0.002127705
filter133⟷filter143	GSX1⟷KDM2B	Homeodomain⟷CxxC	5.75E-06
filter133⟷filter199	GSX1⟷PRKRIR	Homeodomain⟷THAP finger	8.59E-06
filter133⟷filter171	GSX1⟷SOX1	Homeodomain⟷Sox	0.000746888
filter62⟷filter96	HES2⟷FOXN3	bHLH⟷Forkhead	1.48E-05
filter62⟷filter129	HES2⟷GATA5	bHLH⟷GATA	2.18E-10
filter62⟷filter133	HES2⟷GSX1	Homeodomain⟷bHLH	0.002993578
filter62⟷filter160	HES2⟷HHEX	Homeodomain⟷bHLH	0.025163548
filter62⟷filter153	HES2⟷HOXA11	Homeodomain⟷bHLH	1.35E-08
filter62⟷filter175	HES2⟷KDM2B	bHLH⟷CxxC	0.049123556
filter62⟷filter199	HES2⟷PRKRIR	bHLH⟷THAP finger	8.52E-08
filter62⟷filter108	HES2⟷RXRB	bHLH⟷Nuclear receptor	0.006442359
filter62⟷filter159	HES2⟷SOX1	bHLH⟷Sox	0.003489763
filter62⟷filter78	HES2⟷ZKSCAN1	C2H2 ZF⟷bHLH	4.53E-08
filter160⟷filter199	HHEX⟷PRKRIR	Homeodomain⟷THAP finger	0.025461016
filter160⟷filter167	HHEX⟷SOX1	Homeodomain⟷Sox	1.29E-05
filter153⟷filter160	HOXA11⟷HHEX	Homeodomain⟷Homeodomain	0.000478357
filter153⟷filter199	HOXA11⟷PRKRIR	Homeodomain⟷THAP finger	6.69E-09
filter153⟷filter159	HOXA11⟷SOX1	Homeodomain⟷Sox	9.52E-05
filter27⟷filter30	IRX4⟷DMRTA2	Homeodomain⟷DM	4.27E-05
filter27⟷filter96	IRX4⟷FOXN3	Homeodomain⟷Forkhead	4.17E-08
filter27⟷filter129	IRX4⟷GATA5	Homeodomain⟷GATA	0.001458864
filter27⟷filter133	IRX4⟷GSX1	Homeodomain⟷Homeodomain	0.00010058
filter1⟷filter62	IRX4⟷HES2	Homeodomain⟷bHLH	0.028695778
filter27⟷filter160	IRX4⟷HHEX	Homeodomain⟷Homeodomain	0.006852958
filter1⟷filter153	IRX4⟷HOXA11	Homeodomain⟷Homeodomain	0.004003367
filter1⟷filter143	IRX4⟷KDM2B	Homeodomain⟷CxxC	0.02803546
filter27⟷filter198	IRX4⟷PAX2	Homeodomain⟷Homeodomain,Paired box	0.000599869
filter1⟷filter199	IRX4⟷PRKRIR	Homeodomain⟷THAP finger	0.015852369
filter27⟷filter89	IRX4⟷RARB	Homeodomain⟷Nuclear receptor	0.000154095
filter27⟷filter139	IRX4⟷RARG	Homeodomain⟷Nuclear receptor	1.70E-05
filter27⟷filter57	IRX4⟷RFX7	Homeodomain⟷RFX	1.55E-05
filter27⟷filter108	IRX4⟷RXRB	Homeodomain⟷Nuclear receptor	0.000122858
filter27⟷filter35	IRX4⟷SIX4	Homeodomain⟷Homeodomain	0.036570768
filter1⟷filter171	IRX4⟷SOX1	Homeodomain⟷Sox	0.044628918
filter1⟷filter61	IRX4⟷TCF15	Homeodomain⟷bHLH	0.01621677
filter27⟷filter189	IRX4⟷ZKSCAN1	Homeodomain⟷C2H2 ZF	0.044039359
filter47⟷filter84	KDM2B⟷GATA5	CxxC⟷GATA	0.013086664
filter143⟷filter160	KDM2B⟷HHEX	Homeodomain⟷CxxC	0.001874772
filter143⟷filter153	KDM2B⟷HOXA11	Homeodomain⟷CxxC	3.19E-09
filter175⟷filter199	KDM2B⟷PRKRIR	CxxC⟷THAP finger	0.029126468
filter143⟷filter159	KDM2B⟷SOX1	CxxC⟷Sox	0.00015308
filter67⟷filter84	PRKRIR⟷GATA5	THAP finger⟷GATA	7.18E-05

filter57⟷filter96	RFX7⟷FOXN3	RFX⟷Forkhead	0.003548686
filter57⟷filter129	RFX7⟷GATA5	RFX⟷GATA	0.002120495
filter57⟷filter62	RFX7⟷HES2	RFX⟷bHLH	1.43E-05
filter57⟷filter153	RFX7⟷HOXA11	Homeodomain⟷RFX	0.00190261
filter57⟷filter175	RFX7⟷KDM2B	RFX⟷CxxC	0.020444823
filter57⟷filter199	RFX7⟷PRKRIR	RFX⟷THAP finger	4.14E-06
filter57⟷filter108	RFX7⟷RXRB	RFX⟷Nuclear receptor	0.00589543
filter57⟷filter159	RFX7⟷SOX1	RFX⟷Sox	0.023260037
filter57⟷filter61	RFX7⟷TCF15	RFX⟷bHLH	0.01193643
filter57⟷filter189	RFX7⟷ZKSCAN1	RFX⟷C2H2 ZF	0.01226628
filter57⟷filter86	RFX7⟷ZNF202	RFX⟷C2H2 ZF	0.014782827
filter108⟷filter153	RXRB⟷HOXA11	Homeodomain⟷Nuclear receptor	3.88E-06
filter108⟷filter143	RXRB⟷KDM2B	Nuclear receptor⟷CxxC	6.81E-05
filter108⟷filter199	RXRB⟷PRKRIR	Nuclear receptor⟷THAP finger	0.000642153
filter108⟷filter167	RXRB⟷SOX1	Nuclear receptor⟷Sox	1.22E-05
filter10⟷filter104	SIX4⟷DMRTA2	Homeodomain⟷DM	0.004236246
filter10⟷filter80	SIX4⟷DNMT1	Homeodomain⟷CxxC	0.029940625
filter10⟷filter17	SIX4⟷DUXA	Homeodomain⟷Homeodomain	7.44E-05
filter10⟷filter96	SIX4⟷FOXN3	Homeodomain⟷Forkhead	1.06E-10
filter35⟷filter84	SIX4⟷GATA5	Homeodomain⟷GATA	0.009772488
filter10⟷filter133	SIX4⟷GSX1	Homeodomain⟷Homeodomain	1.17E-08
filter10⟷filter62	SIX4⟷HES2	Homeodomain⟷bHLH	9.62E-15
filter10⟷filter160	SIX4⟷HHEX	Homeodomain⟷Homeodomain	5.49E-12
filter10⟷filter153	SIX4⟷HOXA11	Homeodomain⟷Homeodomain	6.55E-17
filter10⟷filter47	SIX4⟷KDM2B	Homeodomain⟷CxxC	0.039227817
filter10⟷filter198	SIX4⟷PAX2	Homeodomain⟷Homeodomain,Paired box	8.25E-06
filter10⟷filter67	SIX4⟷PRKRIR	Homeodomain⟷THAP finger	0.000821242
filter10⟷filter89	SIX4⟷RARB	Homeodomain⟷Nuclear receptor	0.000197954
filter10⟷filter139	SIX4⟷RARG	Homeodomain⟷Nuclear receptor	0.000161492
filter10⟷filter57	SIX4⟷RFX7	Homeodomain⟷RFX	3.80E-06
filter10⟷filter108	SIX4⟷RXRB	Homeodomain⟷Nuclear receptor	2.94E-10
filter10⟷filter23	SIX4⟷SOX1	Homeodomain⟷Sox	0.025635601
filter10⟷filter63	SIX4⟷TCF15	Homeodomain⟷bHLH	0.000833746
filter10⟷filter152	SIX4⟷TLX2	Homeodomain⟷Homeodomain	0.007335701
filter10⟷filter154	SIX4⟷UNKNOWN	Homeodomain⟷UNKNOWN	0.000148137
filter10⟷filter54	SIX4⟷ZFX	Homeodomain⟷C2H2 ZF	0.004371377
filter10⟷filter41	SIX4⟷ZKSCAN1	Homeodomain⟷C2H2 ZF	0.004145332
filter5⟷filter84	SNAI3⟷GATA5	C2H2 ZF⟷GATA	6.57E-06
filter5⟷filter27	SNAI3⟷IRX4	Homeodomain⟷C2H2 ZF	0.026424291
filter5⟷filter10	SNAI3⟷SIX4	Homeodomain⟷C2H2 ZF	0.001231352
filter23⟷filter84	SOX1⟷GATA5	Sox⟷GATA	0.014882266
filter159⟷filter199	SOX1⟷PRKRIR	Sox⟷THAP finger	0.005266152
filter61⟷filter96	TCF15⟷FOXN3	bHLH⟷Forkhead	1.94E-07
filter61⟷filter129	TCF15⟷GATA5	bHLH⟷GATA	7.50E-06
filter61⟷filter133	TCF15⟷GSX1	Homeodomain⟷bHLH	3.17E-05

filter62⟷filter63	TCF15⟷HES2	bHLH⟷bHLH	0.015057894
filter61⟷filter160	TCF15⟷HHEX	Homeodomain⟷bHLH	0.007053379
filter63⟷filter153	TCF15⟷HOXA11	Homeodomain⟷bHLH	0.015061257
filter61⟷filter143	TCF15⟷KDM2B	bHLH⟷CxxC	8.97E-10
filter61⟷filter199	TCF15⟷PRKRIR	bHLH⟷THAP finger	2.49E-06
filter61⟷filter108	TCF15⟷RXRB	bHLH⟷Nuclear receptor	0.003760887
filter61⟷filter159	TCF15⟷SOX1	bHLH⟷Sox	0.024715282
filter129⟷filter152	TLX2⟷GATA5	Homeodomain⟷GATA	0.049552316
filter16⟷filter62	TLX2⟷HES2	Homeodomain⟷bHLH	0.005464262
filter153⟷filter196	TLX2⟷HOXA11	Homeodomain⟷Homeodomain	0.022440048
filter27⟷filter173	TLX2⟷IRX4	Homeodomain⟷Homeodomain	0.048355552
filter16⟷filter143	TLX2⟷KDM2B	Homeodomain⟷CxxC	0.003728609
filter173⟷filter199	TLX2⟷PRKRIR	Homeodomain⟷THAP finger	0.032834049
filter152⟷filter167	TLX2⟷SOX1	Homeodomain⟷Sox	0.017990916
filter16⟷filter61	TLX2⟷TCF15	Homeodomain⟷bHLH	0.003050355
filter78⟷filter100	TLX2⟷ZKSCAN1	Homeodomain⟷C2H2 ZF	0.005676285
filter54⟷filter84	ZFX⟷GATA5	C2H2 ZF⟷GATA	0.000126567
filter78⟷filter96	ZKSCAN1⟷FOXN3	C2H2 ZF⟷Forkhead	1.84E-05
filter129⟷filter189	ZKSCAN1⟷GATA5	C2H2 ZF⟷GATA	0.020347564
filter78⟷filter133	ZKSCAN1⟷GSX1	Homeodomain⟷C2H2 ZF	0.004532009
filter78⟷filter160	ZKSCAN1⟷HHEX	Homeodomain⟷C2H2 ZF	0.000224975
filter41⟷filter153	ZKSCAN1⟷HOXA11	Homeodomain⟷C2H2 ZF	0.002052756
filter41⟷filter143	ZKSCAN1⟷KDM2B	C2H2 ZF⟷CxxC	0.0373158
filter189⟷filter199	ZKSCAN1⟷PRKRIR	C2H2 ZF⟷THAP finger	0.000486019
filter78⟷filter108	ZKSCAN1⟷RXRB	C2H2 ZF⟷Nuclear receptor	0.000848699
filter78⟷filter166	ZKSCAN1⟷SOX1	C2H2 ZF⟷Sox	0.02176073
filter63⟷filter78	ZKSCAN1⟷TCF15	C2H2 ZF⟷bHLH	0.031414495
filter78⟷filter90	ZKSCAN1⟷ZNF202	C2H2 ZF⟷C2H2 ZF	0.009838888
filter2⟷filter30	ZNF202⟷DMRTA2	C2H2 ZF⟷DM	0.035636216
filter86⟷filter96	ZNF202⟷FOXN3	C2H2 ZF⟷Forkhead	0.02631752
filter33⟷filter129	ZNF202⟷GATA5	C2H2 ZF⟷GATA	0.018945943
filter2⟷filter62	ZNF202⟷HES2	C2H2 ZF⟷bHLH	0.007104995
filter90⟷filter153	ZNF202⟷HOXA11	Homeodomain⟷C2H2 ZF	0.010360849
filter27⟷filter144	ZNF202⟷IRX4	Homeodomain⟷C2H2 ZF	0.044577055
filter90⟷filter143	ZNF202⟷KDM2B	C2H2 ZF⟷CxxC	0.01087048
filter90⟷filter199	ZNF202⟷PRKRIR	C2H2 ZF⟷THAP finger	0.005676285
filter10⟷filter148	ZNF202⟷SIX4	Homeodomain⟷C2H2 ZF	0.049931244
filter86⟷filter159	ZNF202⟷SOX1	C2H2 ZF⟷Sox	0.046686771
filter2⟷filter61	ZNF202⟷TCF15	C2H2 ZF⟷bHLH	0.011246917

Table S5: Human promoter unique TF interactions			
filter_interaction	TF_Interaction	Family_Interaction	adjusted_pval
filter133⟷filter178	ARID3B⟷DNMT1	ARID/BRIGHT⟷CxxC	6.86E-12
filter124⟷filter133	DMRTA2⟷DNMT1	DM⟷CxxC	1.11E-14
filter39⟷filter161	DMRTA2⟷EGR1	DM⟷C2H2 ZF	9.98E-06
filter101⟷filter143	DMRTA2⟷HHEX	Homeodomain⟷DM	5.71E-05
filter161⟷filter162	DNMT1⟷EGR1	CxxC⟷C2H2 ZF	0.000187249
filter28⟷filter174	DNMT1⟷HOXA11	Homeodomain⟷CxxC	4.29E-07
filter155⟷filter159	DNMT1⟷KDM2B	CxxC⟷CxxC	0.005490864
filter151⟷filter162	DNMT1⟷LCOR	CxxC⟷Pipsqueak	0.000117664
filter133⟷filter148	DNMT1⟷TET1	CxxC⟷CxxC	4.65E-33
filter28⟷filter57	DNMT1⟷ZNF202	CxxC⟷C2H2 ZF	0.000916891
filter54⟷filter195	DNMT1⟷ZNF263	CxxC⟷C2H2 ZF	0.002379617
filter133⟷filter192	DNMT1⟷ZNF35	CxxC⟷C2H2 ZF	9.40E-13
filter71⟷filter107	EGR1⟷ARID3B	C2H2 ZF⟷ARID/BRIGHT	3.01E-05
filter71⟷filter92	EGR1⟷HMG20B	Sox⟷C2H2 ZF	1.24E-06
filter161⟷filter174	EGR1⟷HOXA11	Homeodomain⟷C2H2 ZF	4.57E-06
filter90⟷filter161	EGR1⟷KDM2B	CxxC⟷C2H2 ZF	0.001643881
filter151⟷filter161	EGR1⟷LCOR	C2H2 ZF⟷Pipsqueak	1.31E-06
filter131⟷filter161	EGR1⟷SNAI3	C2H2 ZF⟷C2H2 ZF	5.24E-08
filter71⟷filter148	EGR1⟷TET1	CxxC⟷C2H2 ZF	2.15E-11
filter71⟷filter195	EGR1⟷ZNF263	C2H2 ZF⟷C2H2 ZF	0.002039419
filter71⟷filter118	EGR1⟷ZNF32	C2H2 ZF⟷C2H2 ZF	3.34E-08
filter78⟷filter101	ENSG00000235187⟷HHEX	Homeodomain⟷Ets	6.55E-06
filter17⟷filter133	FOXK2⟷DNMT1	Forkhead⟷CxxC	5.90E-11
filter17⟷filter161	FOXK2⟷EGR1	Forkhead⟷C2H2 ZF	0.019849637
filter17⟷filter101	FOXK2⟷HHEX	Forkhead⟷Homeodomain	8.81E-06
filter80⟷filter138	FOXK2⟷KDM2B	Forkhead⟷CxxC	9.54E-05
filter21⟷filter80	FOXK2⟷TLX2	Forkhead⟷Homeodomain	0.01682431
filter101⟷filter107	HHEX⟷ARID3B	Homeodomain⟷ARID/BRIGHT	0.000772337
filter162⟷filter197	HHEX⟷DNMT1	Homeodomain⟷CxxC	0.005126141
filter10⟷filter71	HHEX⟷EGR1	Homeodomain⟷C2H2 ZF	0.000478663
filter101⟷filter174	HHEX⟷HOXA11	Homeodomain⟷Homeodomain	1.44E-10
filter151⟷filter197	HHEX⟷LCOR	Homeodomain⟷Pipsqueak	2.19E-10
filter131⟷filter135	HHEX⟷SNAI3	Homeodomain⟷C2H2 ZF	6.81E-09
filter101⟷filter148	HHEX⟷TET1	Homeodomain⟷CxxC	5.55E-24
filter10⟷filter21	HHEX⟷TLX2	Homeodomain⟷Homeodomain	0.022836552
filter10⟷filter102	HHEX⟷ZNF263	Homeodomain⟷C2H2 ZF	3.84E-05
filter101⟷filter118	HHEX⟷ZNF32	Homeodomain⟷C2H2 ZF	1.24E-07
filter135⟷filter192	HHEX⟷ZNF35	Homeodomain⟷C2H2 ZF	1.34E-06
filter92⟷filter133	HMG20B⟷DNMT1	Sox⟷CxxC	7.89E-20
filter92⟷filter101	HMG20B⟷HHEX	Sox⟷Homeodomain	2.20E-07
filter92⟷filter102	HMG20B⟷ZNF263	Sox⟷C2H2 ZF	1.43E-06
filter1⟷filter39	HOXA6⟷DMRTA2	Homeodomain⟷DM	0.000279832
filter1⟷filter54	HOXA6⟷DNMT1	Homeodomain⟷CxxC	0.004288393

filter1⟷filter161	HOXA6⟷EGR1	Homeodomain⟷C2H2 ZF	1.27E-05
filter1⟷filter78	HOXA6⟷ENSG00000235187	Homeodomain⟷Ets	0.00691044
filter1⟷filter197	HOXA6⟷HHEX	Homeodomain⟷Homeodomain	5.43E-06
filter1⟷filter98	HOXA6⟷KDM2B	Homeodomain⟷CxxC	0.007277367
filter1⟷filter151	HOXA6⟷LCOR	Homeodomain⟷Pipsqueak	1.50E-07
filter1⟷filter128	HOXA6⟷LHX5	Homeodomain⟷Homeodomain	0.01607373
filter1⟷filter131	HOXA6⟷SNAI3	Homeodomain⟷C2H2 ZF	0.002589448
filter1⟷filter189	HOXA6⟷SOX1	Sox⟷Homeodomain	0.011781033
filter1⟷filter126	HOXA6⟷TLX2	Homeodomain⟷Homeodomain	0.029590696
filter1⟷filter59	HOXA6⟷ZKSCAN1	Homeodomain⟷C2H2 ZF	0.002400118
filter1⟷filter57	HOXA6⟷ZNF202	Homeodomain⟷C2H2 ZF	1.16E-05
filter1⟷filter195	HOXA6⟷ZNF263	Homeodomain⟷C2H2 ZF	0.000182924
filter1⟷filter118	HOXA6⟷ZNF32	Homeodomain⟷C2H2 ZF	0.0009448
filter33⟷filter133	IRF3⟷DNMT1	IRF⟷CxxC	2.70E-13
filter33⟷filter71	IRF3⟷EGR1	IRF⟷C2H2 ZF	4.36E-07
filter33⟷filter101	IRF3⟷HHEX	IRF⟷Homeodomain	7.17E-06
filter33⟷filter130	IRF3⟷TLX2	IRF⟷Homeodomain	4.30E-07
filter138⟷filter178	KDM2B⟷ARID3B	ARID/BRIGHT⟷CxxC	0.000370208
filter124⟷filter138	KDM2B⟷DMRTA2	DM⟷CxxC	2.27E-05
filter173⟷filter197	KDM2B⟷HHEX	Homeodomain⟷CxxC	0.00165582
filter151⟷filter159	KDM2B⟷LCOR	CxxC⟷Pipsqueak	1.33E-07
filter173⟷filter195	KDM2B⟷ZNF263	CxxC⟷C2H2 ZF	0.038854905
filter138⟷filter192	KDM2B⟷ZNF35	CxxC⟷C2H2 ZF	1.34E-05
filter121⟷filter133	LHX5⟷DNMT1	Homeodomain⟷CxxC	3.80E-12
filter71⟷filter121	LHX5⟷EGR1	Homeodomain⟷C2H2 ZF	8.58E-06
filter19⟷filter101	LHX5⟷HHEX	Homeodomain⟷Homeodomain	0.004020722
filter21⟷filter83	LHX5⟷TLX2	Homeodomain⟷Homeodomain	0.028737386
filter41⟷filter102	LHX5⟷ZNF263	Homeodomain⟷C2H2 ZF	5.73E-06
filter131⟷filter133	SNAI3⟷DNMT1	CxxC⟷C2H2 ZF	5.36E-30
filter131⟷filter138	SNAI3⟷KDM2B	CxxC⟷C2H2 ZF	5.84E-15
filter117⟷filter124	SOX1⟷DMRTA2	Sox⟷DM	0.013489901
filter109⟷filter162	SOX1⟷DNMT1	Sox⟷CxxC	0.005835994
filter73⟷filter161	SOX1⟷EGR1	Sox⟷C2H2 ZF	0.003025464
filter101⟷filter110	SOX1⟷HHEX	Sox⟷Homeodomain	0.003296044
filter160⟷filter174	SOX1⟷HOXA11	Sox⟷Homeodomain	3.04E-06
filter159⟷filter160	SOX1⟷KDM2B	Sox⟷CxxC	0.012152077
filter151⟷filter160	SOX1⟷LCOR	Sox⟷Pipsqueak	5.08E-08
filter109⟷filter125	SOX1⟷LHX5	Sox⟷Homeodomain	0.000158352
filter117⟷filter131	SOX1⟷SNAI3	Sox⟷C2H2 ZF	6.24E-05
filter21⟷filter73	SOX1⟷TLX2	Sox⟷Homeodomain	0.041759796
filter117⟷filter195	SOX1⟷ZNF263	Sox⟷C2H2 ZF	0.000640375
filter160⟷filter192	SOX1⟷ZNF35	Sox⟷C2H2 ZF	2.55E-05
filter0⟷filter162	SOX3⟷DNMT1	Sox⟷CxxC	0.040168612
filter69⟷filter161	SOX3⟷EGR1	Sox⟷C2H2 ZF	7.93E-05
filter0⟷filter101	SOX3⟷HHEX	Sox⟷Homeodomain	1.05E-05

filter1⟷filter180	SOX3⟷HOXA6	Sox⟷Homeodomain	0.017315501
filter69⟷filter160	SOX3⟷SOX1	Sox⟷Sox	0.001305497
filter21⟷filter180	SOX3⟷TLX2	Sox⟷Homeodomain	0.008802917
filter69⟷filter102	SOX3⟷ZNF263	Sox⟷C2H2 ZF	4.12E-05
filter148⟷filter160	TET1⟷SOX1	Sox⟷CxxC	8.61E-16
filter21⟷filter178	TLX2⟷ARID3B	Homeodomain⟷ARID/BRIGHT	0.042530051
filter143⟷filter193	TLX2⟷DMRTA2	Homeodomain⟷DM	0.007554303
filter162⟷filter193	TLX2⟷DNMT1	Homeodomain⟷CxxC	0.029581514
filter48⟷filter161	TLX2⟷EGR1	Homeodomain⟷C2H2 ZF	0.006097183
filter21⟷filter92	TLX2⟷HMG20B	Sox⟷Homeodomain	0.02816662
filter21⟷filter174	TLX2⟷HOXA11	Homeodomain⟷Homeodomain	0.001296995
filter21⟷filter199	TLX2⟷KDM2B	Homeodomain⟷CxxC	0.020374461
filter151⟷filter193	TLX2⟷LCOR	Homeodomain⟷Pipsqueak	7.29E-07
filter21⟷filter131	TLX2⟷SNAI3	Homeodomain⟷C2H2 ZF	1.43E-05
filter21⟷filter148	TLX2⟷TET1	Homeodomain⟷CxxC	3.12E-16
filter21⟷filter59	TLX2⟷ZKSCAN1	Homeodomain⟷C2H2 ZF	0.019110844
filter21⟷filter57	TLX2⟷ZNF202	Homeodomain⟷C2H2 ZF	0.002240944
filter21⟷filter195	TLX2⟷ZNF263	Homeodomain⟷C2H2 ZF	0.0002947
filter118⟷filter193	TLX2⟷ZNF32	Homeodomain⟷C2H2 ZF	0.004170142
filter130⟷filter192	TLX2⟷ZNF35	Homeodomain⟷C2H2 ZF	6.62E-06
filter59⟷filter133	ZKSCAN1⟷DNMT1	CxxC⟷C2H2 ZF	5.11E-16
filter59⟷filter101	ZKSCAN1⟷HHEX	Homeodomain⟷C2H2 ZF	3.90E-06
filter59⟷filter160	ZKSCAN1⟷SOX1	Sox⟷C2H2 ZF	0.002379519
filter57⟷filter71	ZNF202⟷EGR1	C2H2 ZF⟷C2H2 ZF	0.002000326
filter57⟷filter135	ZNF202⟷HHEX	Homeodomain⟷C2H2 ZF	0.021816047
filter57⟷filter174	ZNF202⟷HOXA11	Homeodomain⟷C2H2 ZF	0.000574539
filter57⟷filter90	ZNF202⟷KDM2B	CxxC⟷C2H2 ZF	0.002906239
filter57⟷filter160	ZNF202⟷SOX1	Sox⟷C2H2 ZF	5.26E-06
filter57⟷filter102	ZNF202⟷ZNF263	C2H2 ZF⟷C2H2 ZF	0.000662677
filter102⟷filter107	ZNF263⟷ARID3B	C2H2 ZF⟷ARID/BRIGHT	1.63E-07
filter174⟷filter195	ZNF263⟷HOXA11	Homeodomain⟷C2H2 ZF	3.53E-05
filter102⟷filter151	ZNF263⟷LCOR	C2H2 ZF⟷Pipsqueak	2.48E-11
filter118⟷filter133	ZNF32⟷DNMT1	CxxC⟷C2H2 ZF	5.00E-18
filter118⟷filter138	ZNF32⟷KDM2B	CxxC⟷C2H2 ZF	5.45E-12
filter118⟷filter160	ZNF32⟷SOX1	Sox⟷C2H2 ZF	0.003032589
